# Author’s reply

**Published:** 2010

**Authors:** B Gitanjali

**Affiliations:** *Department of Pharmacology, JIPMER, Pondicherry, India*

Dear Editor,

The authors have stated the editorial was judgmental rather than being open to all the available evidence.[1,2] However, they have not been able to produce any other additional evidence except for reference No. 4, in their letter, which is a single trial published in Turkish.[3] The Turkish trial was not included in the editorial since it is a good practice to never cite what you have not read. The information given in the abstract which was translated in English was far too less to enable me to draw conclusions one way or the other. This means that we have both looked at the same data and the same evidence from the same studies and drawn different conclusions. Let me explain why I believe there is insufficient information to justify the use of valethamate.

The sum total of all studies published on this drug seems to be limited to those referred to in my editorial and the Turkish trial[3] which is extremely meagre, considering that this drug is in the market for nearly three decades. The total number of patients enrolled in all these trials put together is less than 1000 and the number of patients who have received valethamate is approximately one-third of this, which is a very small number considering the wide use of this drug, as suggested.

We need to look at the trial design, the methodology, the statistical standards such as power and the results of the published trials and construct our own conclusions rather than believing the conclusions stated by the authors of the trials at face value. Just because these trials have concluded that valethamate is effective for facilitating cervical dilatation in the first stage of labor it does not necessarily mean that it does.

Using the sample sizes and primary outcome measure given in one of the more recent trials,[4] the *post hoc* power was calculated, which turned out to be 30%. In order to get 80% power, a sample size of 181 patients in each group is needed. Yet, the authors of this trial state that a sample of 50 patients was needed for 90% power! None of the studies mentioned has even 100 patients in any arm. Hence all the trials cited here[3–6] are grossly underpowered.

One of the trials was published in an in-house journal[5] and the authors have used unpaired Student’s *t* test to compare three groups which is inappropriate as mentioned in the editorial. Hence the conclusions are not valid. Another trial mentions the use of Mann-Whitney tests[4] which is also inappropriate to compare three groups.

The conclusions of the abstract of one of the trials states “doses of antibiotics with local instillation of the antibiotics in the wound significantly reduced post-operative infection rate and are safe and feasible.”[5] It is interesting to note that, the objectives, methodology and results do not state anything about the use of antibiotics. Yet, the conclusions of the abstract state this (not the main text). This makes one question the quality of the publication and by default, the quality of the work itself.

The authors of the letter state that ‘no major adverse reactions have been reported till date’. Considering the poor track record of adverse drug reaction reporting in our country, even if there were any, it may go unreported. The drug is not marketed in the developed countries such as the United States of America, Australia or United Kingdom from where most of the adverse drug reaction signals originate. We need to also keep in mind that less than 400 patients have received valethamate in trial conditions. This number is far too small to pick up rare adverse effects. The authors should take into consideration the fact that more number of adverse events were reported in the group which was given valethamate.[5] In this group 81.6% developed transient tachycardia[3] and though the authors may consider it mild and self-limiting, the percentage is large and caution may be needed when this drug is used in places where facilities for monitoring of women in labor are inadequate. However, since the observation of adverse effects was not one of the objectives (even secondary) of any of these trials, more credence to this information cannot be given.

Most of the trials except one are open design and are not blinded. They state randomization has been done but the method of randomization is not stated. Most of the items in the CONSORT check-list are not reported, which by itself is an impediment to assessing quality.

Therefore, I stand by my views that there is no proof of published evidence so far that the drug is useful. The ‘good’ experience of clinicians who have been using it cannot be taken as a benchmark for continuing to prescribe the drug since it comes very low in the hierarchy of evidence. Considering the fact that valethamate is not listed in the Indian Pharmacopoeia, the lack of a standard for testing and technical specifications for procurement should also be borne in mind.
